# Impact of traffic intensity and vehicular emissions on heavy metal content in vineyard soils, grapes, and wine: a comparative study of two vineyards in South Moravia (Czech Republic)

**DOI:** 10.1007/s10653-025-02530-9

**Published:** 2025-05-20

**Authors:** Lubomír Prokeš, Jitka Hegrová, Božena Průšová, Mojmír Baroň, Blanka Hablovičová, Jiří Sochor, Roman Ličbinský

**Affiliations:** 1 Department of Physics, Chemistry and Vocational Education, Faculty of Education, Poříčí 623/7, 603 00 Brno, Czech Republic; 2 Transport Research Centre, Líšeňská 33a, 636 00 Brno, Czech Republic; 3 Faculty of Horticulture, Department of Viticulture and Enology, Mendel University in Brno, Valtická 337, 69144 Lednice, Czech Republic

**Keywords:** Traffic emissions, Heavy metals, Vineyard soil, Grapes, Wine quality, Comparative analysis, Environmental pollution

## Abstract

The primary objective of this study is to evaluate the impact of vehicular traffic emissions on the contamination of wine production by heavy metals. For this purpose, selected heavy elements (As, Ba, Cd, Cr, Cu, Ni, Pb, and Zn) were determined in the samples of vineyard soils, grapes, final wines, and samples from the various phases of the winemaking process of two South Moravian (Czech Republic) vineyards with different intensity of traffic. After the visualisation of the data, the interpretation of the results and risk assessment calculations were performed. The results obtained indicate that contamination of grapes with aerosol is the most important and soil contamination is of minor relevance. The application of fungicides was the primary source of copper and zinc in soils and grapes. During the winemaking process, there is a significant decrease in the content of heavy elements originating from emissions from vehicular traffic. However, winemaking technology was found to be the most important source of heavy elements in the final wine. The health risk assessment indicates a low risk of consumption of both grapes and wine from both the examined and the reference wineries.

**Supplementary Information:**

The online version contains supplementary material available at 10.1007/s10653-025-02530-9.

## Introduction

Transportation and vehicle emissions have a significant impact on the pollution of agricultural soils and plants near roads and highways; however, there are only limited studies on the accumulation of heavy metals in vineyard soils and grapes. Particulate matter released from vehicles in solid and liquid form is a major source of heavy metals (Gupta, 2020; De Silva et al., 2021; Singh et al., 2023). Heavy elements in exhaust emissions from gasoline and diesel fuel combustion,such as lead (Pb), cadmium (Cd), chromium (Cr), nickel (Ni), zinc (Zn), and arsenic (As), are derived from the raw product, but can also be introduced as additives during production or contaminants during storage (Gupta, 2020; Johansson et al., 2000; De Silva et al., 2021). Before the use of leaded gasoline was prohibited in the 1990s, atmospheric deposition was one of the main sources of Pb in roadside plants (De Silva et al., 2021) and in wines (Medina et al., 2000). Historical use of leaded gasoline is an important contributor to Pb contamination, with residual levels still persisting in soils and road dust. Catalytic converters (VEC) emit platinum group elements (Pt, Pd, and Rh) through wear and abrasion. During deterioration, the tyres release Zn, Cd, Cr, Cu, and Pb from additives (such as zinc oxide) used in rubber vulcanisation. Brake linings emit metals such as Cu, Fe, Zn, Sb, Cr, and Pb during braking. Engine components and lubricants are sources of metals such as Cu, Mn, Ni, Pb (from bearings), and Zn (from anti-wear additives) through the engine wear and additives in oils. Asphalt and bitumen on road surfaces release Zn, Cu, Ni, As, and other metals through abrasion and weathering (Ozaki et al., 2004). Metals in vehicular source particulates deposit on roadside soils and vegetation through dry and wet deposition processes. Vehicles can also resuspend dust that has settled on the road, which can contain heavy metals from various sources (Adamiec et al., 2016). Runoff from the road surface can carry metals into adjacent soils and vegetation, particularly during rain events. This runoff mobilises metals from the road surfaces and deposited particulates. In addition to traffic and vehicular emissions, there are other sources of heavy metals that can contribute to the total amount of heavy metals in vineyard soils and grapes.

The primary source of heavy metals in the environment is the lithosphere. Weathering of metal-rich rocks can contribute to background levels of metals such as Ba, Cr, and Ni in soils and dust (Bălc et al., 2018; Milićević et al., 2018). However, this is generally less significant compared to anthropogenic sources (Angon et al., 2024).

Agricultural activities are another source of heavy metals in soils and plants. Phosphate fertilisers release heavy metals, such as Cd, V, Cr, As, etc., into agricultural soils (Angon et al., 2024; Hu et al., 2024). The application of sewage sludge also contributes to the heavy metal content of soils (Pinamonti et al., 1999). The water used for irrigation can also be contaminated with heavy metals (Cd, Cr, Cu, Ni, Pb, Zn, etc.) from various sources (Soleimani et al., 2023; Vystavna et al., 2015; Yang et al., 2022). Copper-based products, such as the Bordeaux mixture (CuSO_4_ + Ca(OH)_2_), blue vitriol (CuSO_4_) or copper oxychloride (CuCl_2_. 3 Cu(OH)_2_), are used as fungicides, for example, to suppress downy mildew in vineyards (Angon et al., 2024; Komárek et al., 2010). Some metals such as Zn, Pb, Cr, Ni, and Cd are present in the mixture as additives (Zn and Mn in the fungicide mancozeb [C_4_H_6_N_2_S_4_Mn]_x_(Zn)_y_; Pořízka et al., 2021) and impurities (Bălc et al., 2018; Mirlean et al., 2005). Sodium arsenite (NaAsO_2_) has also been used in some countries as a fungicide against Esca grapevine disease (Tariba, 2011).

Industrial emissions from factories, power plants, and other facilities can release heavy metals such as lead (Pb), cadmium (Cd), and mercury (Hg) into the atmosphere. These metals can then settle on agricultural lands through precipitation. Industrial site runoff can carry heavy metals into nearby agricultural fields. This runoff may contain metals from various industrial processes, such as metal plating, battery manufacturing, and electronics production (Angon et al., 2024; Moghimi Dehkordi et al., 2024). Heavy metal contamination of vineyard soils in industrial areaa is considerably higher than in comparable vineyard soils in nonindustrial regions (Angelova et al., 1999a, 1999b).

The sources of contamination listed above play a role in the heavy metal content in vineyard soils and vine plants, including grapes. Potentially toxic elements can accumulate in plants through two pathways: absorption into roots from the soil (Milićević et al., 2018; Richardson & Chase, 2021; Sun et al., 2018; Vystavna et al., 2015) and through direct atmospheric deposition of particulate matter onto plant surfaces (leaves and grape skin) (Angelova et al., 1999a, 1999b; Milićević et al., 2018; De Silva et al., 2021). Milićević et al. (2018) found that grape seeds and grapevine leaves accumulate Ba, Cr, Cu, Ni and Zn from the soil, respectively. However, the results of some other authors indicate that Cu (Ko et al., 2007; Richardson & Chase, 2021; Sun et al., 2018) and Zn (Richardson & Chase, 2021) are generally not accumulated from the soil to grape berries. For other heavy elements (As, Cd, Cr and Pb), the accumulation from the soil to the grapes was not proven (Chopin et al., 2008; Ko et al., 2007; Milićević et al., 2018; Richardson & Chase, 2021; Vystavna et al., 2015). Differences in the heavy metal content of different parts of the grape have been found, with the most contaminants in the stems and leaves, less in the skins and seeds, and the least in the pulp (Angelova et al., 1999a, 1999b; Vystavna et al., 2015; De Silva et al., 2021; Yang et al., 2022). This is due to the transfer of different elements from roots to various aerial parts (Chopin et al., 2008; Vystavna et al., 2015) or due to the accumulation of more aerosols, since leaves have relatively large horizontal surface area compared to the grape skin (Angelova et al., 1999a, 1999b; De Silva et al., 2021). The amount of mechanically adhered aerosol pollutants (Cd, Cu, Pb, Zn) can be easily reduced by washing the skins and ramet with water, but the pollutants chemically bound in the vine plant (due to uptake from soil or contamination from aerosol during the development of the berry) cannot be washed away by water (Angelova et al., 1999a).

Winemaking technology is a source of heavy metals in the final wine. Contact with aluminium, brass, stainless steel, and wood winemaking equipment (winemaking machinery, pipes, casks, and barrels), filtration materials (diatomous earth, bentonite), or some additives can lead to heavy metal contamination, for example, Al, Cd, Cr, Cu, Fe, Ni, Pb, Zn (Castiñeira et al., 2004; dos Santos et al., 2019; Dumitriu et al., 2021; Nicolini et al., 2004; Shimizu et al., 2020; Volpe et al., 2009). Acidic conditions in wine can lead to leaching of metals from these materials. In addition to contamination of wine with these agents, the type of wine packaging and storage temperature (Hopfer et al., 2013; Volpe et al., 2009) also affect the elemental profile of wine.

The main objective of this article is to evaluate the impact of traffic intensity and vehicular emissions on heavy metal concentrations in vineyard soils, grapes, and wine, based on a comparison of a highway-proximal vineyard and a reference vineyard not affected by traffic, with respect to other possible sources of metal pollution.

## Materials and methods

### Sample collection

#### Localities

**Fig. 1:**
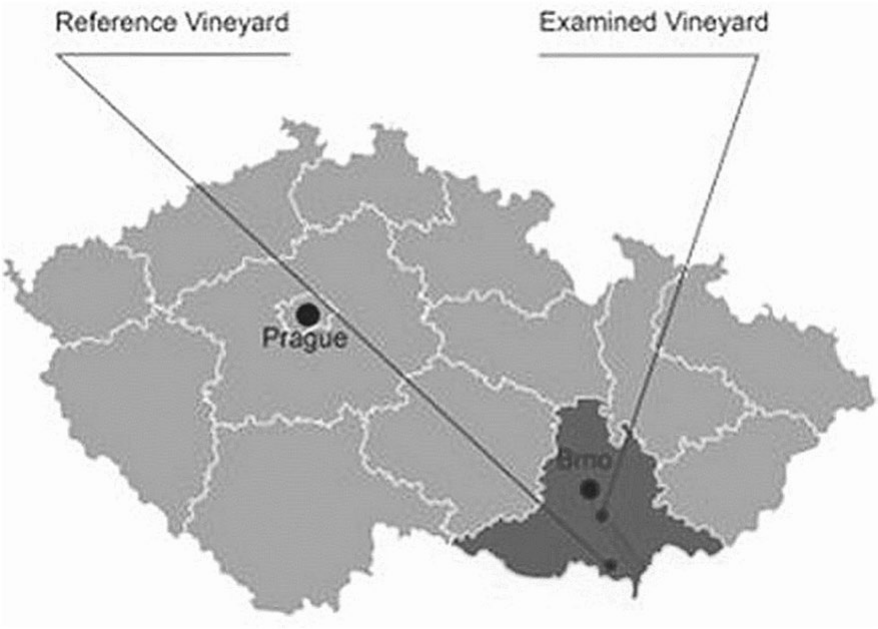
Location of the examined (EXA) and reference (REF) vineyards (South Moravia, Czech Republic)

Both vineyards (Fig. 1) included in this study are situated in the southeast part of the South Moravian region (Czech Republic) with black soil in a Quaternary loess background, far from industrial or energy sources of pollution. No irrigation is applied to either vineyard. Thus, the appropriate site selection has eliminated potential sources of heavy elements, such as bedrock geology, industrial sources, and irrigation.

The vineyard examined (EXA) is located in an area with high traffic intensity due to D2 highway. The intensity of traffic in the vineyard location is on average 24,709 motor vehicles per day. The vineyard boundary is about 70 m from the D2 highway. The samples come from the Punta vineyard track, the cadastral area of Velké Němčice.

The reference vineyard (REF) came from a vineyard on the Hlohovsko vineyard track, cadastral area of Lednice, from an area with very low traffic. The choice of the vineyard for the reference sample was determined by the requirement to minimise the impact of traffic emissions, especially the long distance from busy roads.

#### Soil samples

Soil samples were taken once in both vineyards of interest (EXA and REF). The sample is a mixture of 30 individual samples by a probe sampling rod with an average depth of 0–30 cm. Organic growth was removed before collection. After collection, the puncture samples were always mixed, reduced by quartering, and the aliquot was air-dried at room temperature. Both samples of interest were treated by sieving (2 mm) and grinding (0.63 mm) before extraction.

#### Grape and wine samples

Samples of grapes (Welsh Riesling), as well as other subsequent wine products from EXA vineyard, were provided by the Kamil Prokeš winery, Velké Němčice. The grapes were 30% infested with noble rot, *Botrytis cinerea*; The must had a sugar content of 25° NM (normalised mustmeter). The grapes were ground, destemmed, and prefermentation maceration was performed for 24 h. Subsequently, the must was drained by gravity and fermentation was carried out with the yeast Uvaferm CEG. After the final fermentation, the must was sulphurized to 30 mg.L-1 free SO_2_ and left to age in fine lees. Subsequently, the wine was clarified with Eiwex bentonite at a rate of 150 g.L^−1^ and filtered.

Grape samples (Welsh Riesling), as well as the other subsequent wine products from the REF vineyard, were provided by the DOMAINE EISGRUB Vinné sklepy Lednice winery. The grapes were ground, destemmed and cryomacerated at 10° C for 48 h. The wine was fermented spontaneously, without the use of enzymes or yeast nutrition. After the final fermentation, the coarse sediment was racked on the third day and the wine was left to age on medium and fine lees for another 3 months. Subsequent steps included second racking, clarification with NACALIT bentonite, and filtration with a plate filter with Hobra 15N filter plates (nominal retention 2 μm) and Hobra ST7N filter plates (nominal retention 0.4 μm). The wine was finalised by membrane filtration.

Samples were obtained from the following stages of wine production for both samples analysed: grapes (G), must before sedimentation (MBS), must after sedimentation (MAS), wine after fermentation (WAF), wine before clarification (WBC) and final wine (W). Grape samples (approximately 1 kg) were taken immediately after harvest and frozen. Samples of subsequent products of the gradual wine production were taken by the processors into pre-labelled PET sample bottles, closed, and frozen. The samples were thawed before digestion.

### Chemical analysis

#### Sample digestion

Before use, all containers were soaked in 5% subboiled nitric acid, properly washed with ultrapure water (Millipore, France).

Soil samples were extracted in triplicate in 10 ml of *aqua regia* (2.5 mL of subboiled nitric acid and 7.5 mL of ultrapure hydrochloric acid (Analytica, CZ)) in Teflon tubes in a microwave digestion device (Berghof, Germany) to determine the total content of the selected elements. After digestion, the samples were diluted with ultrapure water to a final volume of 100 mL and diluted 10 times before analysis.

Grapes (with seeds) were dried in a laboratory dryer to a constant weight and crushed in an oscillating ball mill (Retch, Germany). The amount of 0.5 g of homogenised samples was weighed on analytical scales and divided into decomposition Teflon containers, each time 5 repetitions of each sample were prepared.

The wine must and the wine were analysed in a volume of 1.0 mL (1.0 mL ~ 1.0 g) in three repetitions. The material was digested in a mixture of sub-boiled nitric acid (10 mL) and ultrapure hydrogen peroxide (2 mL) (Analytica, CZ) gradually added at doses of 0.5 mL. The samples were left for at least 30 min and then decomposed in the SW-4 microwave digestion device (Berghof, Germany) at a maximum temperature of 230° C and a pressure of 30 bar.

After the digestion cycle, the samples were quantitatively transferred to 25 mL measuring containers and Ultrapure water (Millipore, France) was added to obtain the desired sample volume. Before analysis, the samples were diluted.

#### Tandem mass spectrometry instrumentation

Tandem mass spectrometry (ICP-MS / MS), a sensitive method with low detection limits, was used for the determination of heavy elements in the products. Element determination was performed using a triple quadrupole inductively coupled plasma mass spectrometer (ICPMS/MS Agilent Technologies, Germany) with two quadrupoles (Q1, Q2) and an octopole reaction cell (ORC) under these conditions: the forwarded RF power 1550 W, carrier gas flow rate 1.07 L.min^−1^, integration time per isotope 0.3 s in all modes used. Elements Ba, Cr, Cu, Ni, Pb, and Zn were determined in the He collision gas mode (flow rate 4 ml.min^−1^), Cd was measured in the He / NH_3_ reaction gas mode (flow rate 4 mL.min^−1^ NH_3_ + 1 mL.min^−1^ He), As was determined in the O_2_ reaction gas (flow rate 0.29 ml.min^−1^).

#### Calibration standards, internal standards, and tuning solution

The calibration range 0–100 μg.L^−1^ for all elements was prepared by mixing 1 g.L^−1^ single standard stock solution (Analytika, Czech Republic) in a matrix of 2% subboiled HNO_3_. The internal standard solution (100 μg.L^−1^) was prepared from a commercial 10 mg.L^−1^ mixture of Bi, Ge, In, Li, Sc, Tb, Y (Agilent Technologies, USA) by dilution in 2% HNO_3_. The tuning solution was prepared from a commercial 10 mg.L^−1^ mixture of Li, Co, Y, Ce, Mg, and Tl (Agilent Technologies, USA) at a final concentration of 1 μg.L^−1^, each element diluted in a matrix of 2% HNO_3_. Deionised ultrapure water (Merck Millipore, USA) and subboiled ultrapure HNO_3_ and HCl (Analytika, CZ) were used for the preparation of all solutions.

#### Quality control

The method was evaluated using suitable reference materials:

1. SRM 1640a trace elements in Natural Water (National Institute of Standards & Technology, Gaithersburg, USA) as a material for measuring control (calibration and instrument settings),
2. SRM 1575a—trace elements in Pine Needles (*Pinus taeda*) (National Institute of Standards & Technology, Gaithersburg, USA) as material for control of the entire analysis process.
3. Metranal 33—Matrix reference material—clayey alumina soil with normal level of analytes. (Analytika, CZ) as a material for the control of soil extraction with certified content of leachable *aqua regia* elements.

The limit of detection (LD) and the limit of quantification (LQ) were calculated according to IUPAC from 10 repetitions of the blind test as mean ± 3·SD and mean ± 10·SD, respectively. Measurement uncertainties are determined from two repetitions of the measurements as two times the relative standard deviation of these repetitions and are expressed in percent. The instrument settings and tuning parameters, the recovery of the analytical method, the calibration performance, and the limits of detection and quantification for selected elements are given in the Supplementary Material.

### Assessment of soil, grape, and wine contamination

Data analysis, visualisation and calculations of contamination assessment indices were performed with statistical software R (https://cran.r-project.org). for comparison of differences between elements concentrations of the vineyards via bar plots of elements concentrations and elemental concentration ratios C_EXA_/C_REF_ of the analysed samples. The transfer coefficients C_wine_/C_grapes_ and C_grapes_/C_soil_ for the analysed elements were also visualised via bar plots. The unbiased estimations of means and standard deviations of these ratios were calculated according to Holmes and Buhr (2007). Data visualisation was performed using the *ggplot2* and *ggtheme* R libraries.

The prevention limits of selected heavy elements for Czech agricultural soils (Vácha et al., 2014) were used for comparison of measured concentrations of heavy elements in the vineyard soils.

Soil pollution indices have also been established for the quantification of soil pollution (Liu et al., 2021; Saha et al., 2022; Turhun & Eziz, 2022). C*ontamination factor* (*CF*) was calculated for each chemical element.

$$CF_{i} = {\raise0.7ex\hbox{${C_{i} }$} \!\mathord{\left/ {\vphantom {{C_{i} } {C_{bi} }}}\right.\kern-0pt} \!\lower0.7ex\hbox{${C_{bi} }$}}$$

where *C*_*i*_ is the concentration in the soil, and *C*_*bi*_ is the background reference value for a particular chemical element *i*. As reference values, published data of European agricultural soils (GEMAS) were used (Reimann et al., 2018). Background values for Czech agricultural soils (Skála et al., 2024) were not used, as barium concentrations are not available in this published data set. *Pollution load index* (*PLI*) is calculated as a geometric mean of the contamination factors calculated.

$$PLI = \sqrt[n]{{\mathop \prod \limits_{i = 1}^{n} CF_{i} }}$$

*Ecological risk factor* (*Er*) includes the values of the toxic response factor to the calculation.

$${Er}_{i}= {TR}_{i}\bullet {CF}_{i}$$

where *TR*_*i*_ is the toxic response factor and *CF*_*i*_ is a contamination factor of a particular heavy element. The values of the toxic response factor TR were adopted from the literature (Liu et al., 2021; Saha et al., 2022; Turhun & Eziz, 2022). *Potential ecological risk index* (*RI*) is defined as

$$RI = \mathop \sum \limits_{i = 1}^{n} Er_{i}$$

where *Er*_*i*_ is the ecological risk factor for a particular heavy element. Barium is not included in the calculations of *Er* and *RI* because the toxic response factor for Ba is not available.

The maximum acceptable limit concentrations of selected heavy elements in wine, for comparison with the measured concentrations of heavy elements in the examined wines, were taken from OIV (OIV, 2019; Płotka-Wasylka et al., 2018). For grapes, no acceptable maximum limits are not established.

For both grapes and wine, some parameters of the health assessment were calculated. *Estimated daily intake* (*EDI*, Iwegbue, 2014; Semla et al., 2018; Mirzaei et al., 2020), is calculated (μg.kg_bw_^−1^.day^−1^) with formula

$$EDI = \frac{SFI \cdot MCS}{{BW}}$$

where *SFI* is the mass of the selected dietary ingestion (g.day^−1^), inorganic *MCS* is the concentration of inorganic species in the dietary component (μg.g^−1^ of wet weight), *BW* is the average body weight. For the conversion between the wet weight and the dry weight of the grapes, the factor 0.085 (Mirzaei et al., 2020) was used. Due to the final wine density (0.9912—1.0138 kg.L^−1^; Budziak-Wieczorek et al., 2023), the concentration units ug.L^−1^ and ug.kg^−1^ are interchangeable. The calculated values were compared with the tolerable reference values of dietary intake adapted from the values provided by the European Food Safety Authority (EFSA) (EFSA, 2009, 2010, 2014a, 2014b, 2020, 2022) and Kowalczyk et al. (2022) by dividing by the *BW* values. The tolerable daily intake value for Zn is not established because of Zn low toxicity. *Target hazard quotient* (THQ) is also commonly used in food risk assessment (Iwegbue, 2014; Mirzaei et al., 2020; Naughton & Petróczi, 2008):

$$THQ = \frac{{Efr \cdot ED_{tot} \cdot SFI \cdot MCS}}{RfD \cdot BW \cdot ATn} \cdot 10^{ - 3}$$

where *Efr* is the frequency of exposure (days.year^−1^)*, ED*_*tot*_ is the exposure (years), *SFI* is the mass of selected dietary ingestion (g.day^−1^), *MCS* is the concentration of a particular heavy element in the dietary component (μg.g^−1^ of wet weight), *BW* is the average adult body weight (kg), *RfD* is the oral reference dose (mg.kg^−1^.day^−1^), *ATn* is the averaging time for non-carcinogen (365·number of exposure years, *ED*_*tot*_·365 days), and 10^−3^ is the unit conversion factor. *ED*_tot_ *is calculated* as the mean life expectancy reduced by 18 years (legal drinking age). The safe level is THQ < 1. The oral reference doses *RfD* according to the US Environmental Protection Agency (US EPA) required for the calculation of *THQ* were adopted from the literature (Iwegbue, 2014; Peirovi‑Minaee et al., 2023; Wong et al., 2022) and the USEPA IRIS database (https://iris.epa.gov/AtoZ/). The oral reference dose value *RfD* for Ba is not available. The consumption of grape (4 kg.person^−1^.year^−1^) and wine (18.7 L.person^−1^.year^−1^ or 0.05 L.person^−1^.day^−1^) (*SFI*) values was taken from Chládková et al. (2009), mean body weight (BW) of men (83.6 kg) and women (69.2 kg) body weight (*BW*) from Láchová and Daňková (2010), mean male (75.7 years) and female (81.6 years) at birth from Vrabcová et al. (2017). If the wine consumption parameter is not available, 150 mL.person^−1^.day^−1^ (small wine glass, Shimizu et al., 2020) or 250 mL.person^−1^.day^−1^ (large wine glass, Iwegbue, 2014; Naughton, 2008) can be involved in the calculations. All tables containing original data, calculated and reference values are available in the Supplementary Material.

## Results and discussion

### Impact of vehicular emissions on soil contamination

#### Comparison of heavy metals in soils

**Fig. 2:**
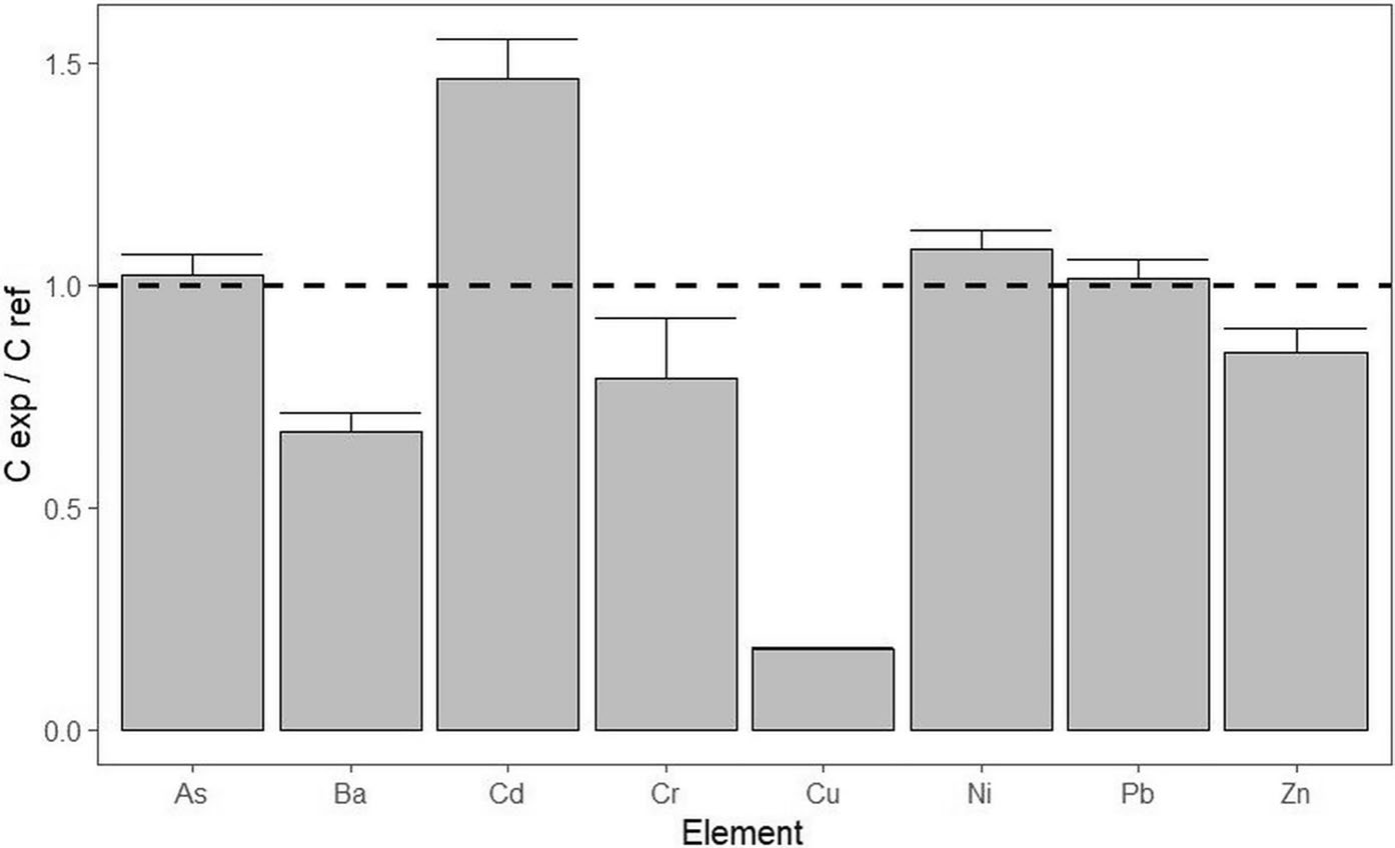
Comparison of heavy element concentrations in the examined vineyard soils (EXA and REF) using CEXA/CREF elemental ratios

Comparison between As, Ba, Cd, Cr, Cu, Ni, and Zn concentrations in the soils of the EXA and reference REF vineyards examined was made using a barplot of elemental concentration ratios C_EXA_/C_REF_ in the analysed soils (Fig. 2). It shows a higher Cd and lower Cu content in EXA vineyard soil, compared to REF. High levels of cadmium in soils (EXA: 0.35 mg.kg^−1^, REF: 0.24 mg.kg^−1^) are probably due to the intensity of traffic, as Cd is contained in brake pads and tyres (Wang & Zhang, 2018). However, soil contamination due to the application of Cd-containing fertilisers (Hu et al., 2024) cannot be excluded. The high level of copper in REF vineyard soil (111 mg.kg^−1^), compared to EXA (20 mg.kg^−1^), is probably due to the application of copper-containing fungicides (Donici et al., 2019; Komárek et al., 2010; Sun et al., 2018; Tariba, 2011; Wang et al., 2023). The concentrations of As, Ni, and Pb are similar in both soils (EXA and the REF), and concentrations of Ba, Cr, and Zn are higher in the REF soil (Fig. 2).

Comparison with published heavy element concentrations in soil samples from South Moravian vineyards (Komárek et al., 2008; Poláková et al., 2023; Pořízka et al., 2021) indicates that both EXA and REF vineyards do not differ significantly from published data, except for Cu concentration in the REF sample. However, this high value is common for vineyards in other regions of the Czech Republic (Ash et al., 2012; Komárek et al., 2010) or in other countries (Komárek et al., 2010).

#### Assessment of soil

The measured concentrations of As, Cd, Cr, Cu, Ni and Zn in soil samples were compared with the prevention limits in agricultural soils in Czech Republic (Vácha et al., 2014). Here, only the copper content is higher (more than twice) than the relevant prevention limit value. The prevention limit for Ba is not established. The *contamination factor* (CF) indicates very strong contamination (CF greater than 5) for the Cu concentration for the reference (REF) vineyard soil, and for the examined (EXA) vineyard soil, the Cu concentration indicates slight contamination (CFI 1–2). The CF values for Cd and Cr concentrations in the examined vineyard soil were close to the threshold value for moderate pollution (CF 2–3), for the reference vineyard, the Cd and Cr soil concentrations were slightly contaminated (CF 1–2). Both localities (EXA and REF vineyards) were slightly contaminated (CF 1–2) for As, Ba and Zn and uncontaminated (CF < 1) for Ni and Pb. According to the calculated *pollution load index* (PLI) (EXA: 1.18, REF: 1.52), both soils were classified as moderately contaminated (PLI = 1–2). According to the values of the *ecological risk factor* (Er) for the Cd examined (EXA), the vineyard soil was classified as moderate (Er = 40–80), for the reference (REF) vineyard sample (REF) as low (Er < 40), but close to the threshold value 40. The concentrations of other elements are of low ecological risk. The values of the *potential ecological risk index* (RI) (EXA: 94.26; REF: 110.92) indicate a low ecological risk (RI < 150) for both soils. Traffic impact, therefore, causes only a low ecological risk. All calculated values related to the soil contamination assessment are available in the Supplementary Material.

### Heavy metal uptake in grapes due to vehicular emissions

#### Comparison of monitored vineyards

**Fig. 3:**
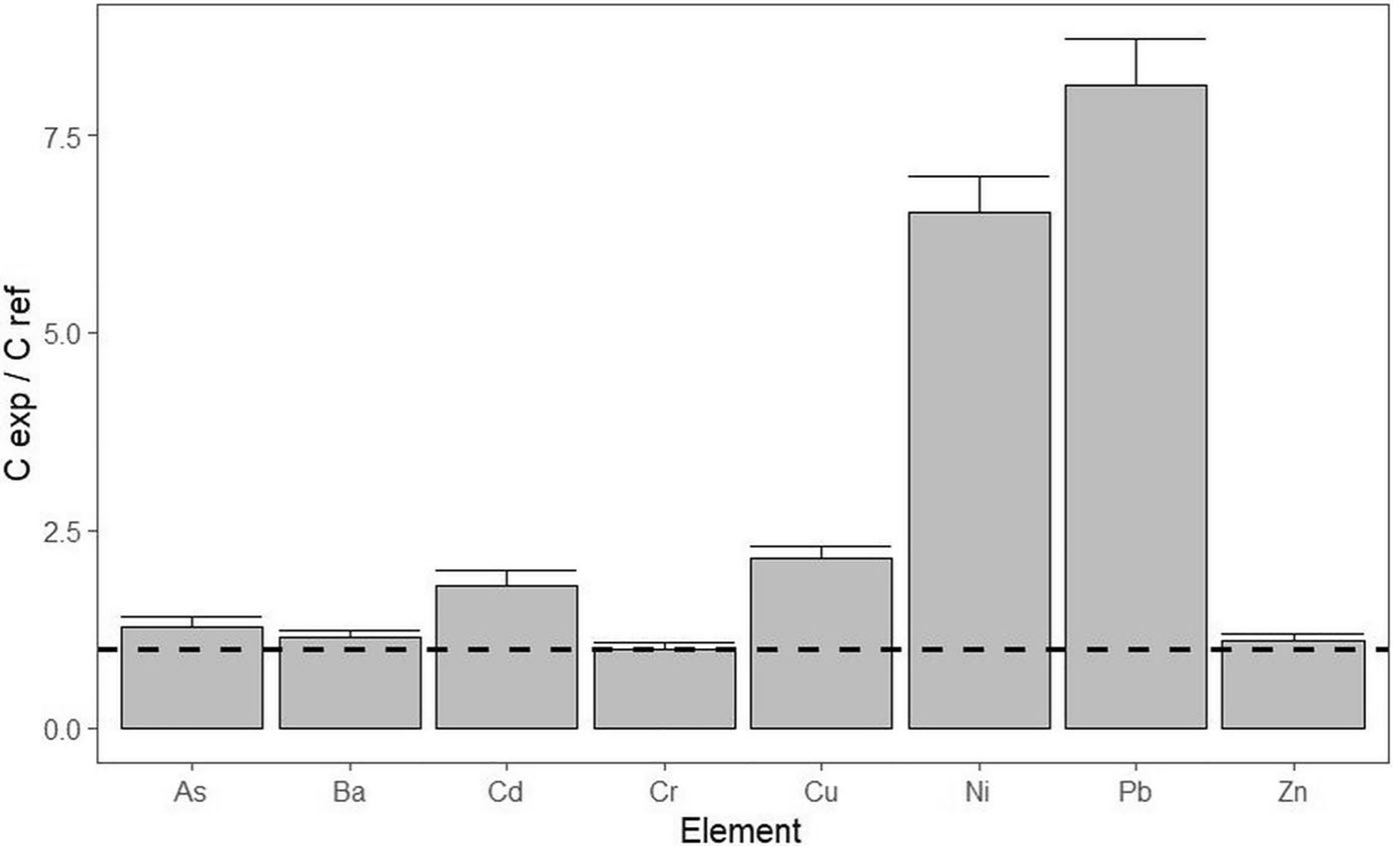
Comparison of heavy element concentrations in examined grapes (EXA and REF) using elemental ratios CEXA/CREF

**Fig. 4:**
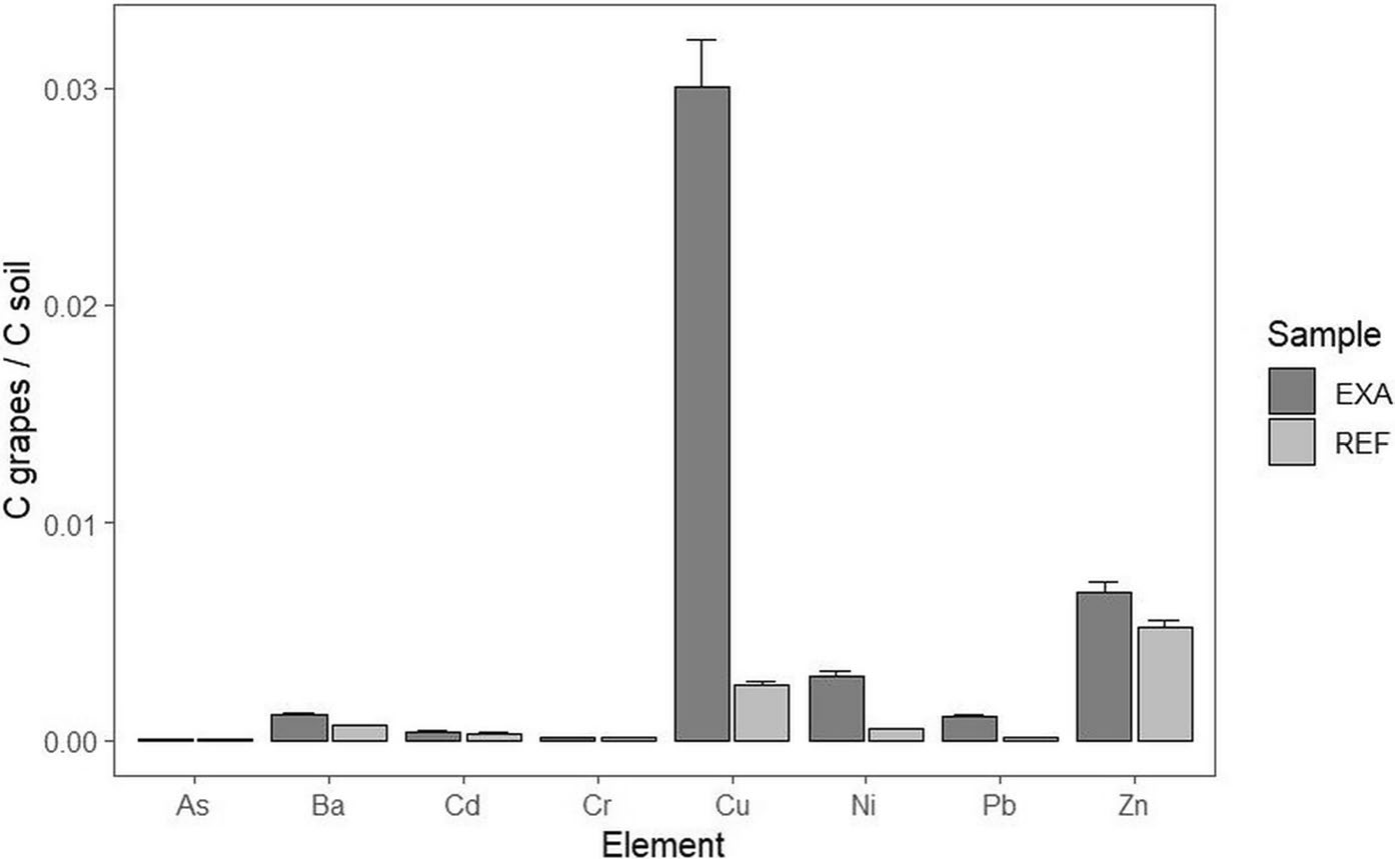
Heavy element concentration ratios Cgrapes/Csoil for examined vineyards (EXA and REF)

The grapes from the examined vineyard (EXA) show higher concentrations of Cd, Cu, Ni, and Pb concentrations, compared to the grapes from the reference (REF) according to the C_EXA_/C_REF_ elemental concentration ratios in the analysed grapes (Fig. 3). It indicates the possible origin of Cd, Ni, and Pb from traffic. Higher concentrations of Cd in both soil and grapes from the examined vineyard indicate its origin from traffic through dust/aerosol particulate matter, as the accumulation of Cd from soil to grapes is not proven (Milićević et al., 2018; Richardson & Chase, 2021). Measured Cd concentrations are significantly lower (Cd: EXA 1.61 ± 0.12 μg.kg^−1^, REF 0.9 ± 0.07 μg.kg^−1^) than the maximum acceptable value of the EU (Commission Regulation, 2023) for the Cd content (30 μg.kg^−1^) in small fruits. Ni and Pb are also of traffic origin, but the deposition of aerosols on grapes is probably the primary source. The origin of Cu is probably from the residues of copper formulations, although traffic cannot be excluded as a source. Differences in the values of the transfer coefficient C_grapes_/C_soil_ for Cu (EXA: 0.35, REF: 0.03) do not indicate the absorption, transport, and accumulation of copper from the vineyard soil to the grapes (see Ko et al., 2007; Sun et al., 2018; Richardson & Chase, 2021) (Fig. 4). The high concentrations of Cu and Zn in both EXA and REF samples probably reflect the application of fungicides such as mancozeb and copper oxychloride (see Pořízka et al., 2021). The low C_grapes_ / C_soil_ ratio of Cu for REF is due to the high concentration of Cu in the soil (compared to EXA). The EXA vineyard also shows higher C_grapes_ / C_soil_ ratios for Ba, Ni, and Pb, probably due to the aerosol origin of these elements. The importance of aerosol contamination of grapes has also been demonstrated for grapes from vineyards in industrial areas (Angelova et al., 1999a, 1999b).

Comparison of the heavy element content in grapes with available published data for grapes (analytical data were obtained for grapes without seeds) from two other South Moravian vineyards without significant traffic impact (Pořízka et al., 2021) indicates a lower content of As and higher levels of Ba, Cd, Cu, Ni, Pb, and Zn in grapes from EXA and REF vineyards. These differences are probably related to vehicular emissions (especially Cd, Ni, and the Pb) and application of fungicides (Cu and Zn). The observed concentrations are lower than in grapes from vineyards in industrial region (Angelova et al., 1999a, 1999b) or published concentrations of heavy elements in grapes from variuos European and non-European countries (see Li et al., 2018; Peirovi‑Minaee et al., 2023).

The heavy element content in grapes may be reduced with prewashing. The washing of grape before processing allows for a decrease in the contents of Pb, Cu, Zn and Cd in the wine without any quality deterioration (Angelova et al., 1999b).

#### Risk assessment for grapes

The *estimated daily intake* (EDI) of heavy elements due to grape consumption is significantly lower than the published values of the tolerable daily intake of EFSA (2009, 2010, 2014a, 2014b, 2020, 2022) and Kowalczyk et al. (2022). Similarly, the *target hazard quotient* (THQ) yields values THQ <  < 1 for all elements in both the EXA and REF samples. Both indicators show the safety of consumption for grapes from both examined localities (EXA and REF). All calculated values related to the grape risk assessment are available in the Supplementary Material.

### Heavy metal content in wines

#### Changes in heavy metal concentrations during winemaking technology

**Fig. 5:**
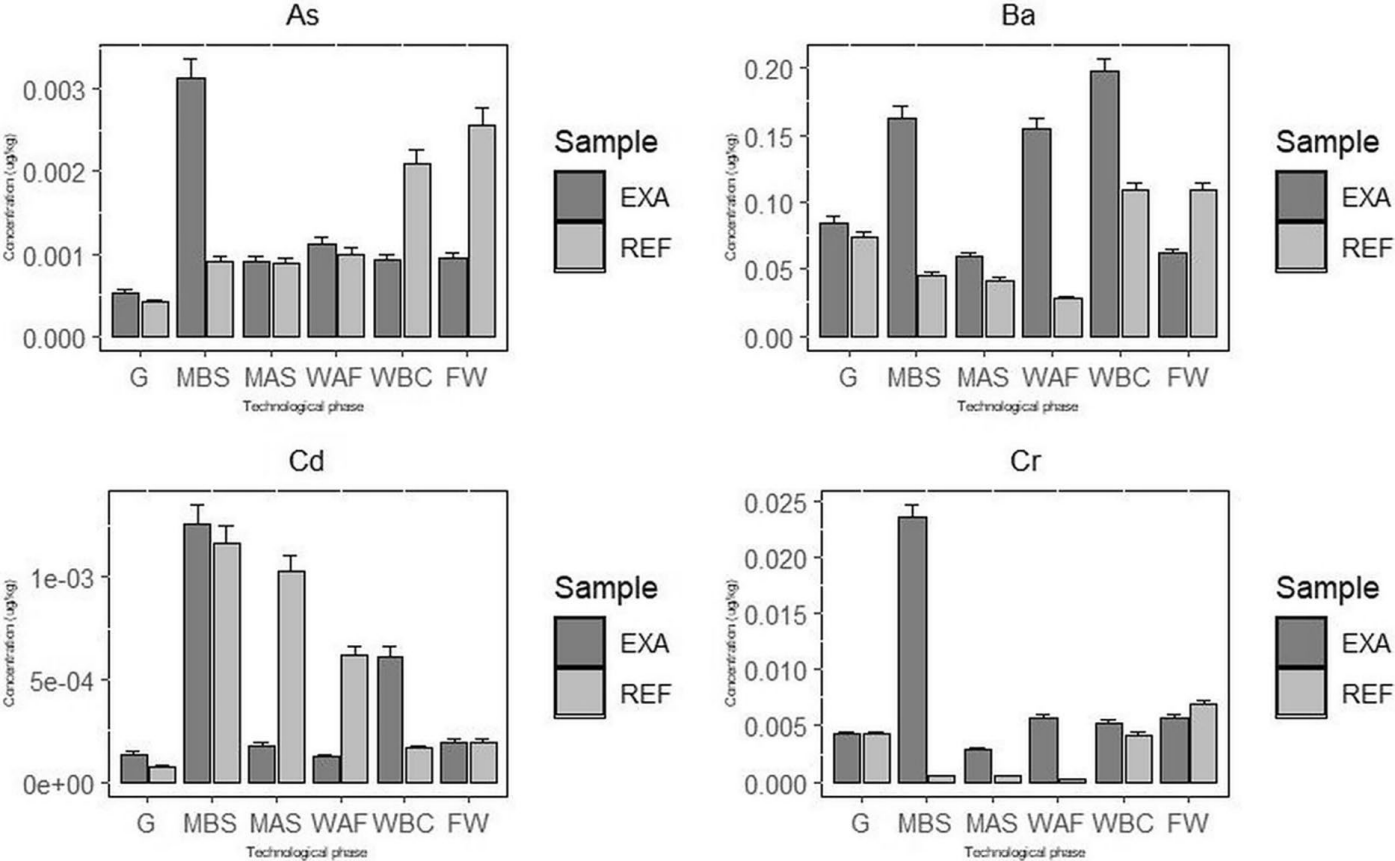
Changes in heavy element concentrations during vine-making technology for both the examined vineyards (EXP and REF) vineyards

Winemaking technology involves several intermediate products between grapes (G) and final wine (W): must before sedimentation (MBS), must after sedimentation (MAS), must after fermentation (WAF), and wine before clarification (WBC). Changes in the concentrations in the course of the vine-making technology are obvious in Fig. 5 and are also reflected in transfer coefficient values (Tab E2s in the Supplementary Material).

Copper concentration decreases dramatically over the course of the winemaking process. In EXA technology, the maximum concentration decrease occurs in the fermentation step (WAF). During fermentation, especially, Cu is generally changed to insoluble sulphides, such as CuS and Cu_2_S, produced by yeast during fermentation and which are removed by sedimentation together with yeast and lees (Castiñeira et al., 2004; Tariba, 2011; Vystavna et al., 2017). The significant decrease in Cu can be explained by coprecipitation with yeast cells during fermentation and/or by forming elemental complexes with tartrates, polyphenols, proteins, and polysaccharides (Dumitriu et al., 2021). For REF technology, the decrease in Cu concentration was slower compared to EXA, and another significant decrease in Cu concentration decrease was observed after the clarification step (WAC), as bentonite clarification usually results in significantly lower Cu levels (Dumitriu et al., 2021; Nicolini et al., 2004). However, prolonged maceration with grape skins can increase Cu concentration in the final wine (Shimizu et al., 2020).

Zinc concentrations gradually decrease during EXA and REF winemaking, especially after fermentation due to precipitation and co-precipitation with organic compounds, which can be easily removed during clarification (Castiñeira et al., 2004; Vystavna et al., 2017), which resulted in significantly lower levels of Zn (Castiñeira et al., 2004; Dumitriu et al., 2021; Nicolini et al., 2004) in the final wine. For EXA, the increase in Zn concentration is negligible in the final wine, possibly due to the addition of bentonite in the clarification step (Castiñeira et al., 2004). For REF, the Zn concentration increases in the last two steps (wine before clarification, WBC, and final wine, W), possibly as a result of zinc-coated equipment and clarification agents. Prolonged maceration with grape skins also increases the concentration of Zn in wine (Shimizu et al., 2020).

Nickel concentrations in both EXA and REF increase in the fermentation step, since stainless steel tanks are often used for fermentation and wine storage (Dumitriu et al., 2021; Tariba, 2011). A jump in Ni concentration for EXA after the fermentation step (WAF) is probably due to the application of SO_2_. For the rest of the wine processing, Ni values decrease, but the addition of bentonite during the fining process is known to increase the Ni concentration (Nicolini et al., 2004;; Shimizu et al., 2020).

Chromium content was significantly reduced in the pressing stage due to an interaction with grape proteins, polyphenols, tartrates and sugars (Tariba, 2011) in the case of REF, but its concentration increased due to the leaching of Cr from steel alloy fermentation vessels (Saha et al., 2022), as for EXA and also REF in the last two steps (wine before clarification, WBC and the final wine, W). Prolonged maceration with grape skins also increases the concentration of Cr in wine (Shimizu et al., 2020).

Lead concentrations decrease during fermentation and the subsequent stages of winemaking. Pb is mainly associated with compounds of relatively low molecular weight and/or high ionic character that created stable complexes but is also complexed by a pectic polysaccharide rhamnogalacturonan (II) that is not degraded during vinification. Only uncomplexed lead could be removed as precipitates of PbS in lees after fermentation (Vystavna et al., 2017). This is obvious in EXA (Pb concentrations are low for REF). For both EXA and REF, Pb concentrations increase before clarification (WBC), probably due to contamination with winemaking equipment, such as containers, pumps, valves, faucets, and tubes (Tariba, 2011; Vystavna et al., 2017). For both EXA and REF, the concentration of Pb decreases during clarification and filtration due to the settle of protein, sulphate and amino acid metal complexes (Dumitriu et al., 2021).

Cadmium concentrations decrease during winemaking for both EXA and REF, because Cd is easily converted to insoluble salts during fermentation (Castiñeira et al., 2004). An increase in Cd concentrations before clarification (WBC) in EXA may be random, because of the low Cd concentration.

Barium concentration commonly decreases during the winemaking process, especially after fermentation, as in REF. The changes in Ba concentration in EXA and the increase in concentration for REF before clarification (WBC) are very difficult to interpret. The addition of clarification agents, such as bentonite, can lead to a slight increase in concentration (Castiñeira et al., 2004; Nicolini et al., 2004; Shimizu et al., 2020).

Arsenic concentration is low (REF) or decreases (EXA) during winemaking and then increases before clarification (WBC), which is very difficult to interpret. The addition of bentonite during the fining process can increase the concentration of As (Nicolini et al., 2004; Shimizu et al., 2020).

**Fig. 6:**
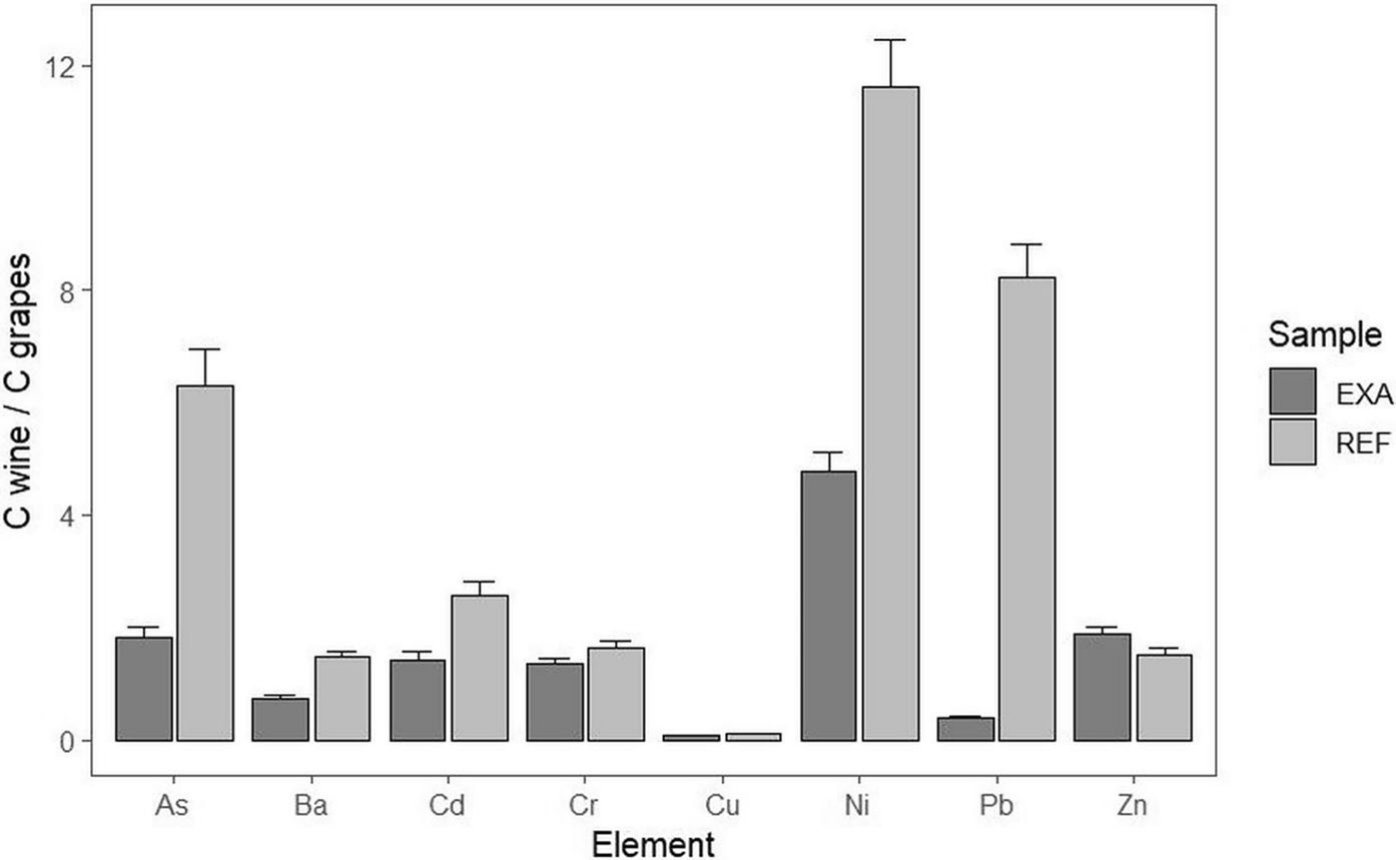
Heavy element concentration ratios Cwine/Cgrapes for examined vineyards (EXA and REF)

It can be inferred that the majority of heavy metals precipitate into the sediment during the fermentation step, even if variations in concentrations can also be influenced by the volatility of the process. For wine from the region impacted by industrial pollution, Angelova et al. (1999b) came to a similar conclusion. For all elements except zinc, REF wines have a higher transfer coefficient (C_wine_ / C_grapes_; Fig. 6). This indicates that EXA winemaking is superior to REF in terms of eliminating heavy metals.

#### Comparison of heavy metal concentrations of wines from monitored vineyards

**Fig. 7:**
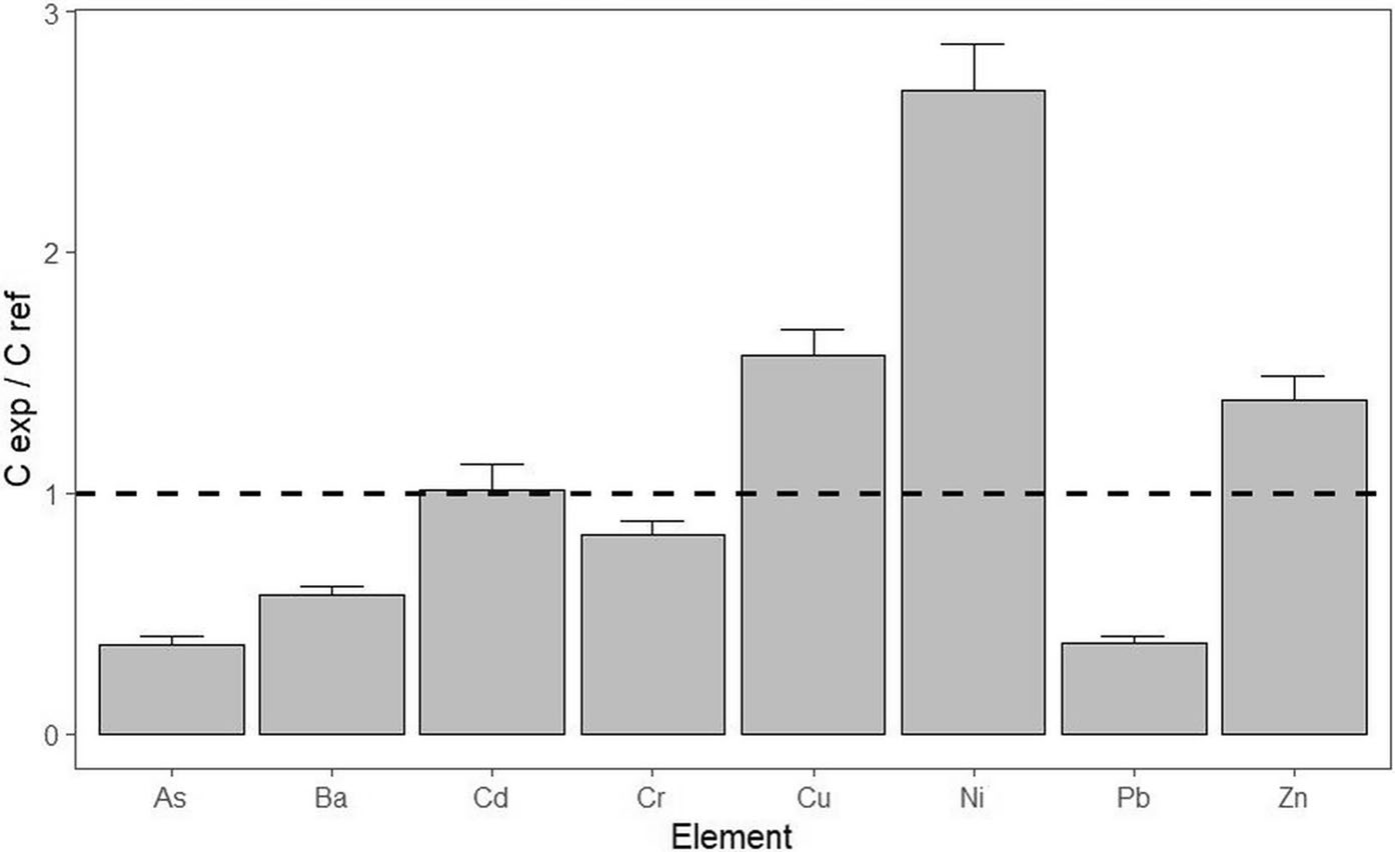
Comparison of the concentrations in the wine of the examined vineyards (EXP and REF) using CEXA/CREF elemental ratios

The heavy element content in the final wine combines the influence of heavy metals on the grapes and wine contamination during the winemaking technology. Therefore, the impact of traffic and vehicular emissions is biased with heavy elements originating from wine technology and storage. Comparison of EXA and REF wines with elemental ratios C_EXA_/C_REF_ for the analysed wines shows that Cu, Ni, and Zn concentrations in the final EXA wine are significantly higher than those of the REF wine, in contrast to the higher content of Pb, As, Ba, and Cr in the REF wine. The Cd concentrations are the same for the EXA and REF wines (Fig. 7). Compared to published values of heavy element concentrations in South Moravian wines (Mlček et al., 2018; Pořízka et al., 2021) and wines from other European countries (Papageorgiou et al., 2023), the concentrations of Ba, Ni and Pb are higher in both EXA and REF wines.

#### Assessment of heavy metals risk for wine

The concentrations of As, Cd, Cu, Pb, and Zn in wine samples from EXA and REF vineyards are lower than the maximum acceptable limits established by the OIV (OIV, 2019; Płotka-Wasylka et al., 2018). The *estimated daily intake* (EDI) values are predominantly lower than the tolerable daily intake values published by the European Food Safety Authority (EFSA) for heavy elements in wines from EXA and REF vineyards. The values of the *target hazard quotient* (THQ) are notably less than 1 for all elements in both the EXA and the REF samples. All calculated values related to the wine risk assessment are available in the Supplementary Material.

## Conclusions

The primary source of heavy metals in soils and grapes from examined (EXA and REF) vineyards is aerosol and dust contamination resulting from vehicle emissions and traffic. In addition to sources of traffic pollution, the application of fungicides constitutes another significant source of heavy elements (Cu and Zn). The phenomenon of contamination by transfer from the soil to the grapes is of minor importance. A substantial proportion of heavy elements, including Ba, Cd, Ni, and Pb, which are known to be associated with traffic pollution, are eliminated during the winemaking process. The primary sources of heavy elements in wine are contamination from winemaking equipment and clarification agents. It has been demonstrated that EXA technology is more effective than REF for the removal of heavy elements from wine. The values of the estimated daily intake and the total hazard quotient are indicative of the safety of consumption of the grapes and wine from the examined vineyards. The grapes and wine from the EXA and REF vineyards are deemed safe for consumption.

## Supplementary Information

Below is the link to the electronic supplementary material.

Supplementary file1 (XLSX 46 kb)

## Data Availability

Datasets are available in the Supplementary material.
